# Relapsed Chronic Lymphocytic Leukemia Heralded by Parathyroid Hormone Related Peptide‐Mediated Hypercalcemia

**DOI:** 10.1002/ccr3.71181

**Published:** 2025-10-12

**Authors:** Srikar Tallavajhala, Joanna Lee, Sean C. Dougherty, Brett R. Kurpiel, Craig A. Portell

**Affiliations:** ^1^ Department of Medicine University of Virginia Health System Charlottesville Virginia USA; ^2^ Division of Hematology/Oncology, Department of Medicine University of Virginia Health System Charlottesville Virginia USA; ^3^ Department of Pathology University of Virginia Health System Charlottesville Virginia USA

**Keywords:** chronic lymphocytic leukemia, hypercalcemia, parathyroid hormone‐related protein, small lymphocytic lymphoma

## Abstract

Hypercalcemia is a common, serious complication in patients with malignancy and is associated with significant morbidity. Compared to patients with solid tumor malignancies, the reported incidence of hypercalcemia in patients with indolent lymphoid malignancies is lower and rare in those with chronic lymphocytic leukemia/small lymphocytic lymphoma (CLL/SLL). In this report, we describe the case of a patient with a history of CLL/SLL previously treated with multiple lines of therapy and thought to be in remission who presented with symptomatic, severe hypercalcemia. Her calcium initially improved; however, it increased after discontinuation of steroids despite aggressive fluid resuscitation, calcitonin, and bisphosphonate therapy. Following an extensive laboratory and radiographic evaluation, her hypercalcemia was concluded to be secondary to elevation in serum parathyroid hormone‐related peptide in the setting of recurrent CLL/SLL. Notably, the patient had no evidence of Richter transformation and initially responded well to steroids, denosumab, and CLL‐directed therapy; however, she later had refractory CLL/SLL and hypercalcemia. Hematologic malignancy, including CLL/SLL, could be considered in the differential diagnosis of hypercalcemia and can be present either at the time of initial diagnosis or signal disease relapse in the appropriate context.

## Graphical abstract

Recurrent chronic lymphocytic leukemia/small lymphocytic lymphoma (CLL/SLL) presenting with severe parathyroid hormone related peptide‐mediated hypercalcemia. Bone marrow and paravertebral biopsies confirmed relapse without evidence of Richter's transformation.

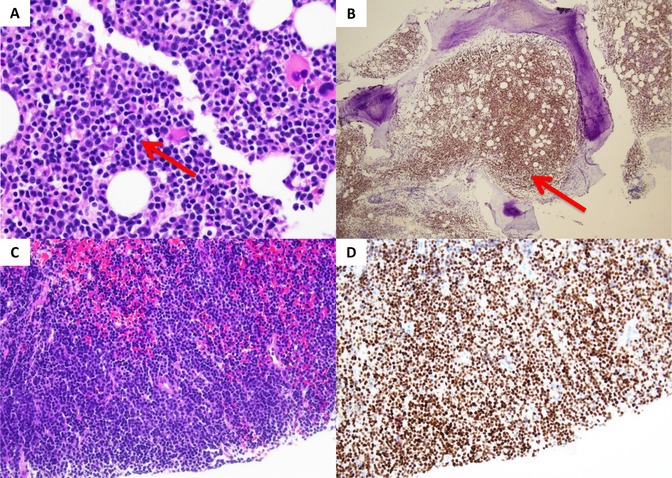

**Summary**

- Hypercalcemia is a commonly encountered electrolyte abnormality in outpatient and hospitalized patients with multiple different causes.
- In cases of parathyroid hormone‐related peptide mediated hypercalcemia, consider indolent lymphoid malignancies in the differential diagnosis.
- In patients with chronic lymphocytic leukemia specifically, parathyroid hormone‐related peptide mediated hypercalcemia can signal relapse of disease.

## 1 Introduction

Hypercalcemia can occur through a number of different mechanisms and is a well‐known sequela of malignancy, occurring in 20%–30% of all cancers [1, 2, 3]. The etiologies of hypercalcemia in malignancy include direct invasion of the bone by tumor, excess 1,25‐dihydroxyvitamin D (calcitriol) production, and secretion of systemic humoral factors resulting in calcium resorption from bone, the latter termed humoral hypercalcemia of malignancy (HHM) [1, 4]. HHM is primarily mediated by parathyroid hormone‐related peptide (PTHrP) produced by tumor cells and can result in abrupt, often severe elevations in serum calcium levels to greater than 13 mg per deciliter (mg/dL). Despite its negative effects observed in the context of malignancy, PTHrP has physiologic roles in endochondral bone formation in embryonic development and also bone remodeling [5].

HHM is commonly observed in several solid tumors, including non‐small cell lung cancer and breast cancer, in addition to multiple hematologic malignancies, including adult T‐cell leukemia/lymphoma and other non‐Hodgkin lymphomas (NHL) [6, 7, 8, 9, 10, 11, 12]. Hypercalcemia in NHL is mediated through conversion of 25‐hydroxyvitamin D to its active metabolite, 1,25‐dihydroxyvitamin D, via the enzyme 1α‐hydroxylase [11, 12]. PTHrP‐mediated hypercalcemia has rarely been described in chronic lymphocytic leukemia/small lymphocytic lymphoma (CLL/SLL), though it has been shown to often be associated with Richter's transformation [13, 14, 15, 16, 17, 18]. Previous reports describing HHM in the context of CLL/SLL without aggressive transformation are limited [19, 20].

In this clinical case report, we describe the evaluation, diagnosis, and management of severe hypercalcemia in a patient with CLL who was ultimately found to have PTHrP‐mediated HHM heralding relapse of her disease without evidence of aggressive transformation.

## 2 Case Presentation

A 63‐year‐old woman with a history of CLL/SLL presented to our tertiary care medical center with a chief complaint of fatigue. She reported rapid progression of fatigue, associated weakness, abdominal discomfort, confusion, melancholy, and left thoracic back pain over the preceding 24 h. Three days prior to her current presentation, she had been discharged from an outside hospital following a brief admission for progressive fatigue and weakness in the setting of severe hypercalcemia. During that admission, she was found to have a calcium level of 14.8 mg/dL and was treated with intravenous fluids, calcitonin, and pamidronate with subjective improvement in her symptoms and downtrend in her calcium to 9.7 mg/dL. She was discharged with plans for outpatient endocrinology follow‐up for a presumed endocrinologic cause of her hypercalcemia at that time.

Regarding her CLL/SLL, she was diagnosed with Rai stage 0, del11q, immunoglobulin heavy‐chain variable region (IGHV) unmutated CLL approximately 6 years prior to the current presentation and initially did not require therapy. Within 1 year of diagnosis, she progressed to Rai stage 3 disease, at which point she underwent six cycles of bendamustine and achieved a remission for 2 years. After progression, she was treated with venetoclax and rituximab. After she was noted to have a new right iliac lesion on positron emission tomography scan about 1 year after initiation of venetoclax and rituximab, a biopsy was obtained and was consistent with CLL. She received radiation therapy to the lesion and was started on acalabrutinib, 100 mg by mouth twice daily. She subsequently stopped acalabrutinib 1 year later due to poor tolerance and disease progression, at which point she was restarted on bendamustine and rituximab again and received six subsequent cycles of therapy. She was not on active therapy at the time of her current presentation to our institution, shortly after finishing bendamustine and rituximab, as it was felt that her disease was in remission. Her other medical history included cervical cancer treated 30 years prior with definitive surgery, chemotherapy, and radiation. She did not have any relevant family, surgical, or social history.

On physical exam, her vital signs were all within normal limits. She appeared fatigued, did not have palpable lymphadenopathy or hepatosplenomegaly, and her abdomen was non‐tender to palpation throughout. Cardiac exam revealed a normal heart rate and regular rhythm without murmurs, rubs, or gallops. She was breathing comfortably on room air and her lungs were clear to auscultation bilaterally. Her skin examination was within normal limits. She did not have any paraspinal tenderness to palpation. She was alert and oriented to person, place, and time. Initial laboratory testing was notable for a white blood cell count of 10.23 k/μL, with differential indicating 72% neutrophils and 19% lymphocytes, hemoglobin of 11.2 g/dL, and platelets of 198 k/μL. Her complete metabolic panel revealed a blood urea nitrogen of 12 mg/dL, creatinine of 0.8 mg/dL, and calcium of 15.1 mg/dL; see Table 1 for complete laboratory results.

**TABLE 1:** Complete laboratory analysis.

Test	Result	Reference range
Complete blood count		
White blood cells (×10^9^/L)	10.2	4–11,000
Hemoglobin (g/dL)	11.22	12.0–16.0
Hematocrit (%)	32.9	35.0–47.0
Platelets (×10^3^/L)	198	150–450
Differential (absolute)		
Neutrophils (×10^9^/L)	6.67	1.8–8.0
Lymphocytes (×10^9^/L)	4.31	1.0–5.0
Monocytes (×10^9^/L)	0.71	0.0–1.0
Eosinophils (×10^9^/L)	0.00	0.0–0.6
Basophils (×10^9^/L)	0.00	0.0–0.2
Complete metabolic panel		
Sodium (mmol/L)	136	136–145
Potassium	3.2	3.4–4.8
Chloride	100	98–107
Bicarbonate	25	23–31
Blood urea nitrogen (mg/dL)	12	10–20
Creatinine	0.8	0.6–1.1
Calcium	15.2	8.5–10.5
Ionized Calcium	7.0	4.4–5.5
Magnesium	1.6	1.6–2.6
Phosphorous	2.8	2.6–4.7
Alkaline phosphatase (U/L)	93	40–150
AST (U/L)	69	< 35 U/L
ALT (U/L)	38	< 55 U/L
Total bilirubin (mg/dL)	0.3	0.3–1.2
Miscellaneous		
25 Hydroxy Vitamin D (ng/mL)	50	20–80
1,25‐Dihydroxy Vitamin D (pg/mL)	71	18–78
PTH (pg/mL)	11.5	9.0–77.0
PTHrP (pmol/L)	8.7	< 4.2
TSH (mIU/L)	1.76	0.45–4.50

Abbreviations: ALT, alanine aminotransferase; AST, aspartate aminotransferase; PTH, parathyroid hormone; PTHrP, parathyroid hormone‐related protein; TSH, thyroid stimulating hormone.

## 3 Differential Diagnosis, Investigations and Treatment

The differential diagnosis associated with hypercalcemia is broad and includes a number of endocrinologic, malignant, dietary, and medication‐related causes. Endocrinologic causes include primary hyperparathyroidism, hyperthyroidism, adrenal insufficiency, and familial hypocalciuric hypercalcemia. The initial branch point in the diagnostic algorithm for hypercalcemia is whether or not the parathyroid hormone (PTH) level is elevated, normal, or low. Our patient's PTH returned at 11.5 pg/mL, which was towards the lower limit of normal and considered appropriately low in this case. This was not consistent with primary hyperparathyroidism, which would result in an elevated PTH despite hypercalcemia. It should also be noted that the severe hypercalcemia observed in this patient would be uncommon in primary hyperparathyroidism, which is usually associated with mild hypercalcemia. A thyroid‐stimulating hormone was obtained and within normal limits, ruling out hyperthyroidism. In the absence of a family history of hypercalcemia, familial hypocalciuric hypercalcemia was felt to be unlikely. The patient did not have any abnormal dietary habits and did not endorse consuming abnormal amounts of calcium or calcium‐containing supplements. She was not prescribed a thiazide diuretic, lithium, calcium, vitamin D, or vitamin A, and thus medications were not suspected as the underlying cause of her presentation.

**FIGURE 1:**
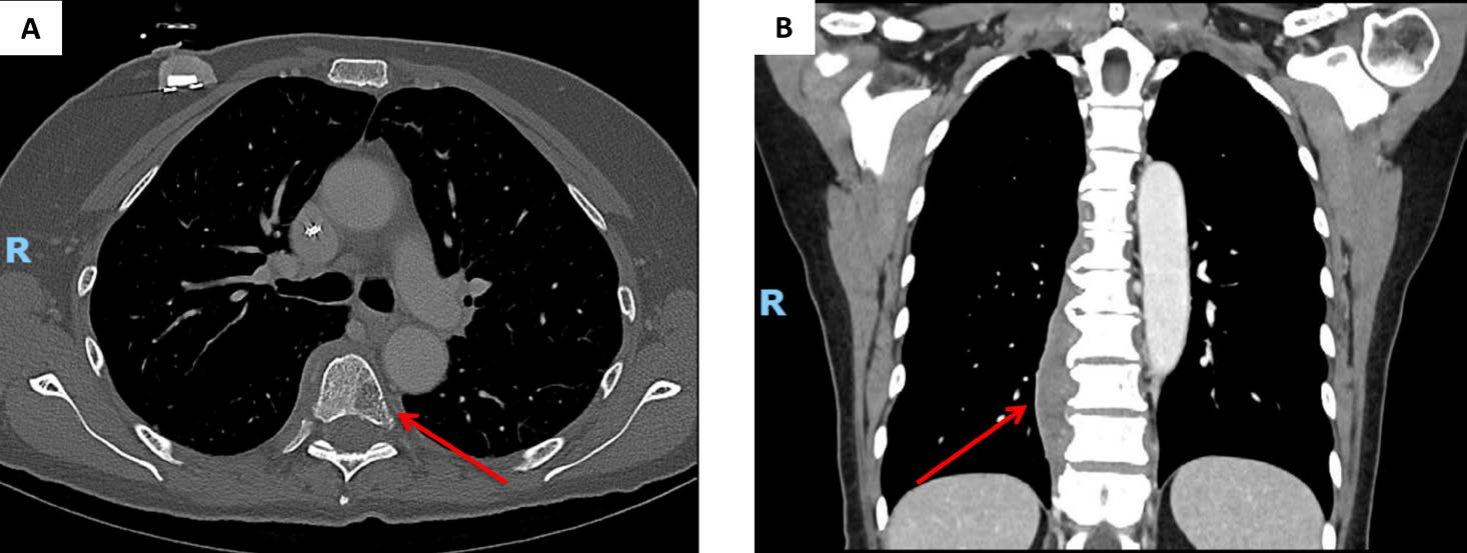
(A) Axial plane computed tomography scan of the chest demonstrating a 1.7 × 1.2‐cm lytic focus within the T6 vertebral body (red arrow). (B) Coronal plane computed tomography scan of the chest showing a right‐sided paravertebral mass measuring 13.6 cm in the craniocaudal dimension spanning from the T5‐6 level to T12. CT‐guided FNA of this mass would later confirm involvement of the patient's CLL/SLL consistent with recurrence.

Causes of hypercalcemia related to malignancy include osteolytic bone metastases, which can occur in multiple different types of cancer, osteoclast‐induced bone resorption from multiple myeloma, PTHrP production, and increased production of 1,25–dihydroxyvitamin D, the latter most commonly observed in lymphoma [1, 21]. Cases of ectopic PTH secretion secondary to ovarian carcinoma, small cell lung cancer, and papillary thyroid cancer have also been described [6, 22, 23]. 25‐hydroxyvitamin D and 1,25–dihydroxyvitamin D levels were obtained and both returned within normal limits at 50 ng/mL and 71 pg/mL, respectively. PTHrP obtained was elevated 8.7 pmol/L. Given the observed elevation in PTHrP, there was concern for underlying malignancy, and computed tomography (CT) scans of the chest, abdomen, and pelvis were obtained. These were notable for diffuse osteolysis in the femur, bones of the pelvis/sacrum, and the spine (Figure 1A) as well as a right‐sided paravertebral soft tissue mass spanning from the T5 to T12 levels and measuring 13 cm in length (Figure 1B). Pathologic lymphadenopathy and/or splenomegaly are present in approximately 50% of cases of symptomatic CLL/SLL and are consistent with at least Rai Stage I or greater [24, 25]. Given the lack of lymphocytosis, lymphadenopathy, or splenomegaly, progression of CLL/SLL was initially felt to be less likely in this patient. No findings consistent with classic presentations for other malignancies were identified either.

**FIGURE 2:**
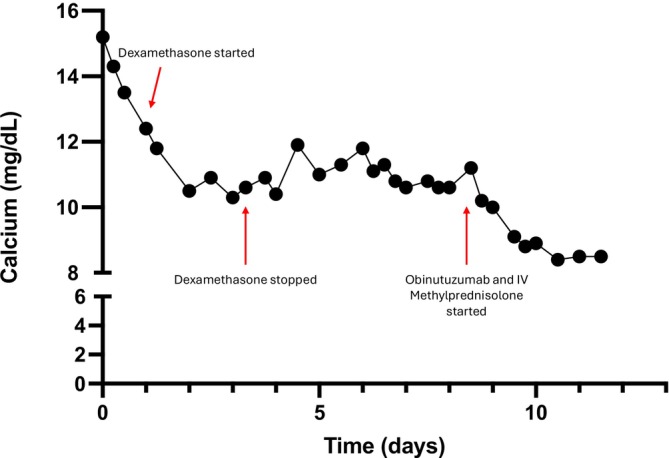
Calcium trend from time of admission until time of discharge. Red arrows indicate times of dexamethasone initiation and discontinuation as well as obinutuzumab and methylprednisolone initiation.

For management of the hypercalcemia, she was started on aggressive intravenous fluid hydration (normal saline at 200 mL per hour), calcitonin, 4 mg/kg administered intramuscularly every 12 h for three doses, and pamidronate, 90 mg injected intravenously over 2 h. Due to initial concern for lymphoma, she was started on dexamethasone, 10 mg by mouth daily on hospital day two, which was continued for 2 days. Following discontinuation of steroids, she then had a significant, rapid rise in her calcium level again from an initial nadir of 10.3 mg/dL back to 11.9 mg/dL (Figure 2). She was subsequently also started on denosumab, 120 mg administered intravenously, which has been shown to be an effective agent in bisphosphonate‐refractory hypercalcemia, likely due to the suppression of PTHrP‐mediated bone resorption [26, 27]. Serum protein electrophoresis and urine protein electrophoresis were performed to evaluate for multiple myeloma or another plasma cell dyscrasia given her osteolytic lesions and were unremarkable, arguing against these entities.

**FIGURE 3:**
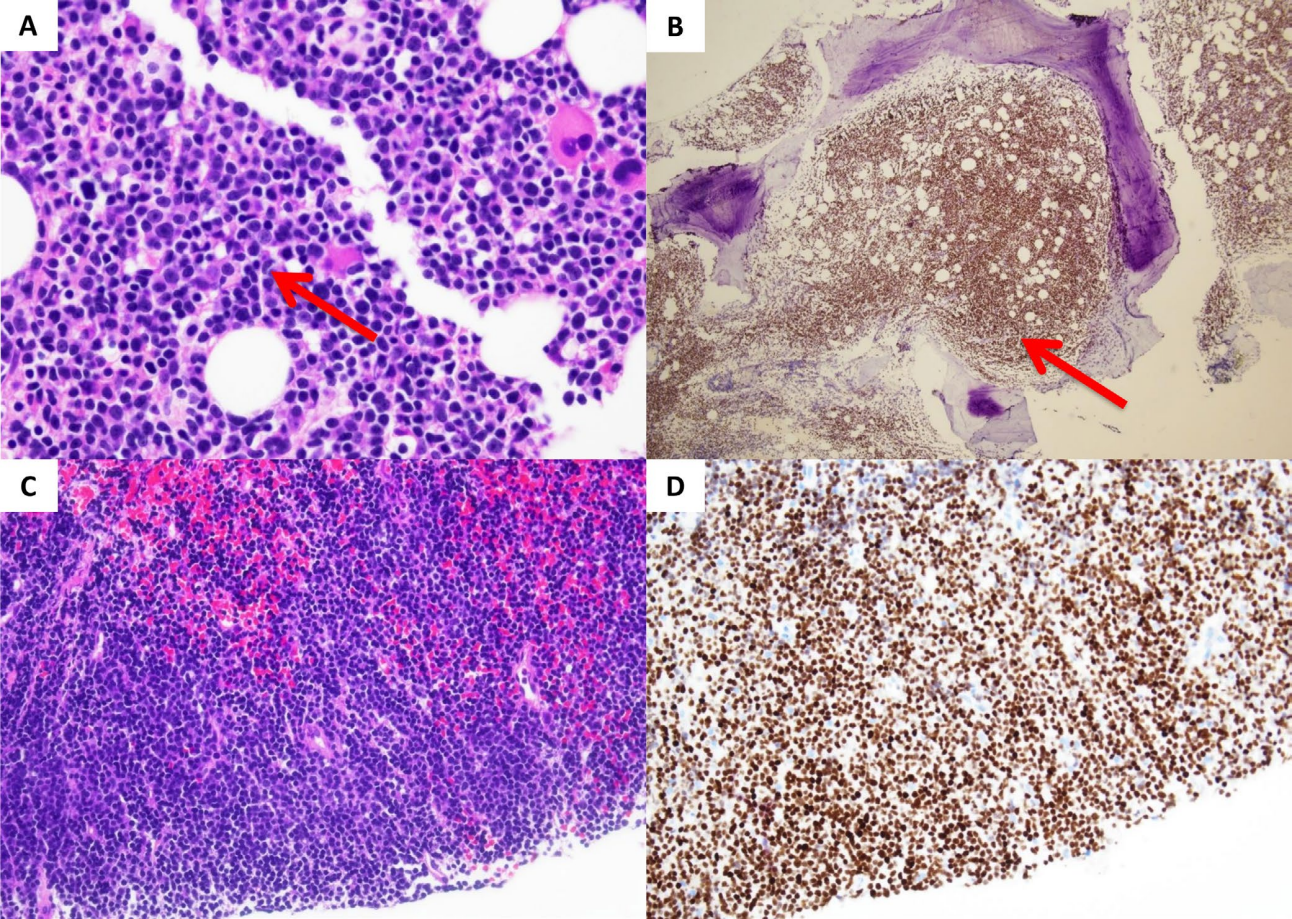
(A) High‐power image showing hypercellular (for age) bone marrow with diffuse involvement by small, monotonous lymphocytes. Scattered megakaryocytes, erythroid precursors, and myeloid precursors are seen in the background (H&E, 400× original image). (B) Low, scanning power image of a LEF1 immunohistochemical stain demonstrating the immense degree of marrow involvement by the patient's known CLL/SLL (20× original image). (C) Microscopic examination of the paravertebral soft tissue mass shows sheets of monotonous, small lymphocytes with coarse chromatin and round nuclei that lack atypia with few to no large forms (H&E, 200× original image). (D) The abnormal lymphocytes were positive for CD20, CD5, CD23, and strong LEF1 (as shown in panel B) by immunohistochemistry, confirming involvement by CLL/SLL (200× original image).

The patient then underwent a CT‐guided fine needle aspiration of the paravertebral mass, which revealed lymphoid tissue involved by a diffuse lymphoid infiltrate with immunohistochemical stains and flow cytometry demonstrating a lambda monotypic cluster differentiation (CD) 5+ B cell population (Figure 3C,D). A bone marrow biopsy was obtained and revealed a hypercellular marrow with diffuse involvement by small, monotonous lymphocytes (Figure 3A,B); flow cytometry of the bone marrow aspirate revealed a lambda monotypic CD5+ B cell population. Neither the biopsy of the paraspinal mass nor the bone marrow biopsy demonstrated any evidence of transformation to an aggressive lymphoma.

## 4 Outcome and Follow‐Up

Overall, her presentation was favored to represent relapse of her CLL with resultant PTHrP‐mediated hypercalcemia, which also resulted in the rapid recurrence of severe hypercalcemia within 3 days of discharge from the outside hospital. Given that the patient had previously been treated with multiple lines of therapy, including a bruton tyrosine kinase inhibitor and a B‐cell leukemia/lymphoma‐2 inhibitor, after a multidisciplinary tumor board discussion, she was started on obinutuzumab via an infusion ramp‐up. High‐dose methylprednisolone, 1000 mg IV daily was also restarted and continued for 3 days. Her serum calcium decreased significantly to 8.5 mg/dL by the time of discharge and remained within normal limits at outpatient follow‐up. She was started on duvelisib, a small molecule inhibitor of the gamma delta isoform of phosphoinositide 3‐kinase, 25 mg by mouth twice daily at that time and continued monthly obinutuzumab. She remained on this therapy for 6 months; however, subsequently had evidence of disease progression on repeat positron emission tomography (PET) scan with extranodal and bone lesions and standardized uptake values all under 7. A serum calcium level obtained shortly after her PET scan was again elevated at 11.9 mg/dL. She was evaluated for chimeric antigen receptor T cell therapy for refractory CLL/SLL; however, she was deemed not to be a candidate in the setting of her poor functional status. Similarly, she was not a candidate for allogeneic stem cell transplant. She was started on pirtobrutinib thereafter; however, she tolerated treatment poorly with nausea, vomiting, and dehydration and stopped after 13 days of therapy. Following a discussion with the patient and her family and in the setting of worsening hypercalcemia, she later transitioned to hospice.

## 5 Discussion

Hypercalcemia is one of the most common metabolic complications of malignancy, estimated to affect up to 30% of patients with cancer [28]. This report describes a case of CLL recurrence heralded as severe, symptomatic hypercalcemia. Despite the presence of new osteolytic lesions, the major driver of this patient's hypercalcemia was felt to be HHM mediated by ectopic PTHrP production. This is a rare paraneoplastic syndrome in patients with CLL that has not been described often in the literature prior to this report [15, 19, 20]. The specific biological mechanisms in CLL/SLL leading to the rarity of PTHrP‐mediated hypercalcemia in this disease are not currently known.

Calcium is a key regulator of multiple physiological processes, and imbalances can lead to systemic symptoms, including abdominal pain, changes in bowel movements, arrhythmias, nephrolithiasis, altered mental status, and seizures [21]. Multiple mechanisms of hypercalcemia in patients with malignancy have been described. HHM, the most common, is caused by the release of PTHrP by tumors. This is most often observed in solid tumor malignancies, particularly squamous cell carcinomas, and is less common in patients with indolent lymphoid malignancies [29]. While the exact mechanism underlying this upregulation is unknown, implicated oncogenes include *TPR‐MET* and *RAS* as well as the NF‐kB and MAPK signaling pathways [30, 31, 32]. Osteolytic metastases with local cytokine release, production of calcitriol by tumor cells, and ectopic secretion of PTH are the other classic causes of HHM. Calcitriol production is a common cause of malignancy‐associated hypercalcemia in patients with lymphoma and other hematologic malignancies [33]. HHM is relatively rare in patients with NHL. Within hematological malignancies, PTHrP mediated hypercalcemia is most associated with adult T‐cell leukemia/lymphoma [9, 10].

Cases of hypercalcemia occurring in patients with CLL have been described, but are rare, which makes identifying the mechanism of disease challenging [18, 29]. Case reports with confirmed elevations in PTHrP are exceedingly rare, and in many cases, the patient had evidence of Richter transformation, which our patient did not. There is a published case of CLL with a confirmed elevation in PTHrP that demonstrated improvement with denosumab [15]. While less common, our report supports the notion that PTHrP‐mediated hypercalcemia as a consequence of CLL/SLL or its recurrence should be considered in the appropriate clinical context. More broadly, although classically associated with solid tumor malignancies, PTHrP‐mediated hypercalcemia due to underlying hematologic malignancy should also be considered in the differential diagnosis associated with hypercalcemia. Acute management of hypercalcemia in these cases should be done with standard therapies, including intravenous fluids, calcitonin (in the absence of renal dysfunction), and anti‐resorptive medications. However, in order to prevent recurrence of hypercalcemia in the context of hematologic malignancy, therapies addressing the underlying disease may need to be utilized.

In summary, we report a rare case of HHM mediated by PTHrP in a patient with CLL/SLL. Unlike other reported cases, our patient had no evidence of Richter transformation and initially responded to steroid therapy. This case demonstrates that hypercalcemia can be seen in CLL/SLL without aggressive transformation.

## Author Contributions

**Srikar Tallavajhala:** methodology, writing – original draft, writing – review and editing. **Joanna Lee:** investigation, writing – original draft, writing – review and editing. **Sean C. Dougherty:** conceptualization, investigation, methodology, writing – original draft, writing – review and editing. **Brett R. Kurpiel:** resources, writing – original draft, writing – review and editing. **Craig A. Portell:** conceptualization, writing – review and editing.

## Ethics Statement

All procedures followed were in accordance with the ethical standards of the responsible committee on human experimentation and with the Helsinki Declaration of 1975, as revised in 2000. This study did not require IRB approval and was not part of a registered clinical trial.

## Consent

Written informed consent was obtained from the patient prior to manuscript composition. All patient protected health information has been anonymized, and no identifiable information has been included in this case report.

## Conflicts of Interest

Craig A. Portell: Consulting: BeiGene, Janseen/Pharmacyclics, Kite, TG Therapeutics, AbbVie, Merck, AstraZeneca. Research funding: Merck, AbbVie, Roche/Genentech, Acerta/AstraZeneca, BeiGene, Kite, Prelude. All authors declare no conflicts of interest.

## Data Availability

Data pertaining to this case report are available upon request from the corresponding author.
